# Comedication that increase the risk of cisplatin-induced hearing loss in pediatric cancer patients: a narrative literature review and meta-analysis

**DOI:** 10.3389/fonc.2026.1769234

**Published:** 2026-05-13

**Authors:** Kim E. de Jager, Nienke Streefkerk, Alexander E. Hoetink, Harm van Tinteren, Martine van Grotel, Marry M. van den Heuvel-Eibrink

**Affiliations:** 1 Princess Máxima Center for Pediatric Oncology, Utrecht, Netherlands; 2 Department of Otorhinolaryngology and Head & Neck Surgery, University Medical Center Utrecht, Utrecht University, Utrecht, Netherlands; 3 Division of Child Health, Wilhelmina Children’s Hospital, Utrecht, Netherlands

**Keywords:** chemotherapeutics, childhood cancer, cisplatin, comedication, hearing loss, ototoxicity, supportive care

## Abstract

**Background:**

Cisplatin induced hearing loss (CIHL) affects 50-70% of all cisplatin-treated children. Cisplatin containing regimens often include various supportive care drugs and chemotherapeutic agents. No structured overview is available of the potential impact of concomitant medication, administered alongside cisplatin, on the increased risk of CIHL.

**Methods:**

A narrative review, based on a PubMed (Medline), EMBASE, and Cochrane CENTRAL search, was conducted to summarize the effect of concomitant chemotherapeutic agents and supportive care drugs on developing hearing loss in cisplatin-treated pediatric cancer patients. The studies were categorized according to type of comedication.

**Results:**

Our review identified 27 relevant studies with a total of 7007 patients. There is heterogeneity among the included studies with respect to design and quality. Nevertheless, the results of the meta-analyses showed that vincristine (OR: 3.80, 95% CI: 2.86-5.04), furosemide (OR: 1.63, 95% CI: 1.14-2.33), aminoglycosides (OR: 1.72, 95% CI: 1.14-2.60) and vancomycin (OR: 1.63, 95% CI: 1.09-2.45) were recurrently found to be associated with an increased risk of CIHL.

**Conclusion:**

This review represents the first comprehensive overview of evidence on the contribution of comedication to the risk of CIHL. Vincristine, furosemide, ahminoglycosides, and vancomycin are associated with an increased risk of CIHL in pediatric cancer patients.

## 1 Introduction

Each year, nearly 400,000 children worldwide are diagnosed with cancer (1, 2). Outcome has increased over the previous decades as a result of improved patient stratification, refined therapeutic options and combinations, and enhanced supportive care regimens (3). Consequently, rising survival rates have raised attention to early and late side effects. An example of a severe, irreversible, and early side effect is cisplatin-induced hearing loss (CIHL) (4). This phenomenon is caused by apoptosis of outer hair cells in the cochlea, due to DNA crosslinking and generation of reactive oxygen species (5). Cisplatin dose contributes to a higher risk of CIHL (6–8). In children, CIHL negatively affects speech development and educational progress, leading to delayed development of social, academic, and emotional skills (9). CIHL may be accompanied by tinnitus and vertigo, thereby contributing to a patient’s lifelong impaired quality of life (4). CIHL affects 50-70% of all cisplatin treated children and is irreversible (4, 10–12).

Cisplatin containing treatment regimens are usually administered in highly aggressive cancer types (13). It is often combined with other chemotherapeutic agents in multi-agent regimens targeting the tumor, including vincristine, doxorubicin, cyclophosphamide, ifosfamide, etoposide, methotrexate, 5-fluorouracil, and lomustine (14). In addition, supportive care typically includes antibiotics, antiemetics, diuretics, and antifungal agents. Loop diuretics, or alternatively mannitol, are used to accomplish forced diuresis. Some of these compounds, such as vincristine, aminoglycosides, glycopeptides, and loop diuretics, have been suggested to be independently ototoxic (4, 15–22). The additive effect of these potential ototoxic agents on CIHL has been studied less extensively.

To our knowledge, a structured overview of the impact of comedication on the risk of CIHL is currently lacking. Therefore, this study summarized the data from all available studies on comedication that may increase the risk of developing CIHL in the pediatric cancer setting. The included meta-analysis provides a quantitative summary and aims to pave the way for universal guidelines managing comedication and better audiological monitoring in pediatric cancer patients receiving cisplatin.

## 2 Methods

### 2.1 Search strategy

We performed literature searches in PubMed (Medline), EMBASE, and Cochrane CENTRAL up to August 22, 2025, using the search strategy and terms as listed in **Supplementary Table 1**. The search strategy included comedication, which comprise both relevant chemotherapeutic agents and supportive care drugs, commonly prescribed alongside cisplatin according to treatment protocols.

### 2.2 Inclusion and exclusion criteria

This review included all pediatric studies that reported on CIHL and that included concomitant chemotherapy and supportive care medication in the analysis. Specifically, we selected studies based on the following inclusion criteria: 1) 80% of the patient cohort was younger than 19 years at diagnosis, 2) the patients received cisplatin treatment, 3) administration of comedication is described in the study, 4) audiological assessments were performed at least at the end of treatment, and 5) absolute numbers or odds ratios were reported regarding hearing loss between the yes/no comedication groups. Studies with less than 20 participants, no differences in use of comedication between two study arms, adult studies, and studies without audiological information were excluded from our review. Reviews, commentaries, (conference) abstracts, and articles in languages other than English were excluded.

### 2.3 Study selection and data extraction

The titles and abstracts of all publications were screened by a single reviewer (K.E.d.J.). In case of uncertainty, inclusion of the study was discussed with the second reviewer (M.M.v.d.H.-E.). Articles selected for full-text screening were reviewed for the following data items: author and year of publication, study design, cohort characterizations (age, sex, disease), in- and exclusion criteria. Data on the effect of the comedication associated with CIHL were collected based on the variety of outcomes in available studies, i.e., prevalences, odds ratio (OR) or hazard ratio (HR). Data extraction was performed by two independent reviewers (K.E.d.J. and N.S.). The included studies were categorized in the following subgroups: chemotherapeutic agents, loop diuretics, and antibiotics (aminoglycosides and glycopeptides). The types of hearing loss assessment and classification system were extracted from the included studies, as this may influence the reported percentages of hearing loss. If multiple ORs are reported in a study, based on different classification systems, the OR from the classification system with the highest concordance with other systems used in studies of that comedication subgroup is included in the meta-analysis (based on Table 5 in Clemens et al. (2019) (23)). Concordance of CTCAE is based on version 4.03, however, similar concordance is assumed for the different CTCAE versions. Furthermore, the cumulative dose of cisplatin was included in the review, as this is known to be associated with the risk of CIHL (4, 6–8). The risk of bias in the included studies was assessed using the QUIPS tool (24).

### 2.4 Analysis

R (4.4.0) and R Studio (“meta” package) were used for the meta-analyses. The studies were categorized by comedication type. The ORs were summarized in forest plots for each comedication investigated in at least three studies. Random-effects models (DerSimonian and Laird method) were employed to pool the ORs and corresponding confidence intervals, accounting for between-study heterogeneity. To assess this heterogeneity, we calculated the Cochran’s Q statistic, the I^2^ statistic, and the between-study variance (Tau^2^) (25, 26). Funnel plots were analyzed for possible bias in the meta-analyses.

## 3 Results

**Figure 1:**
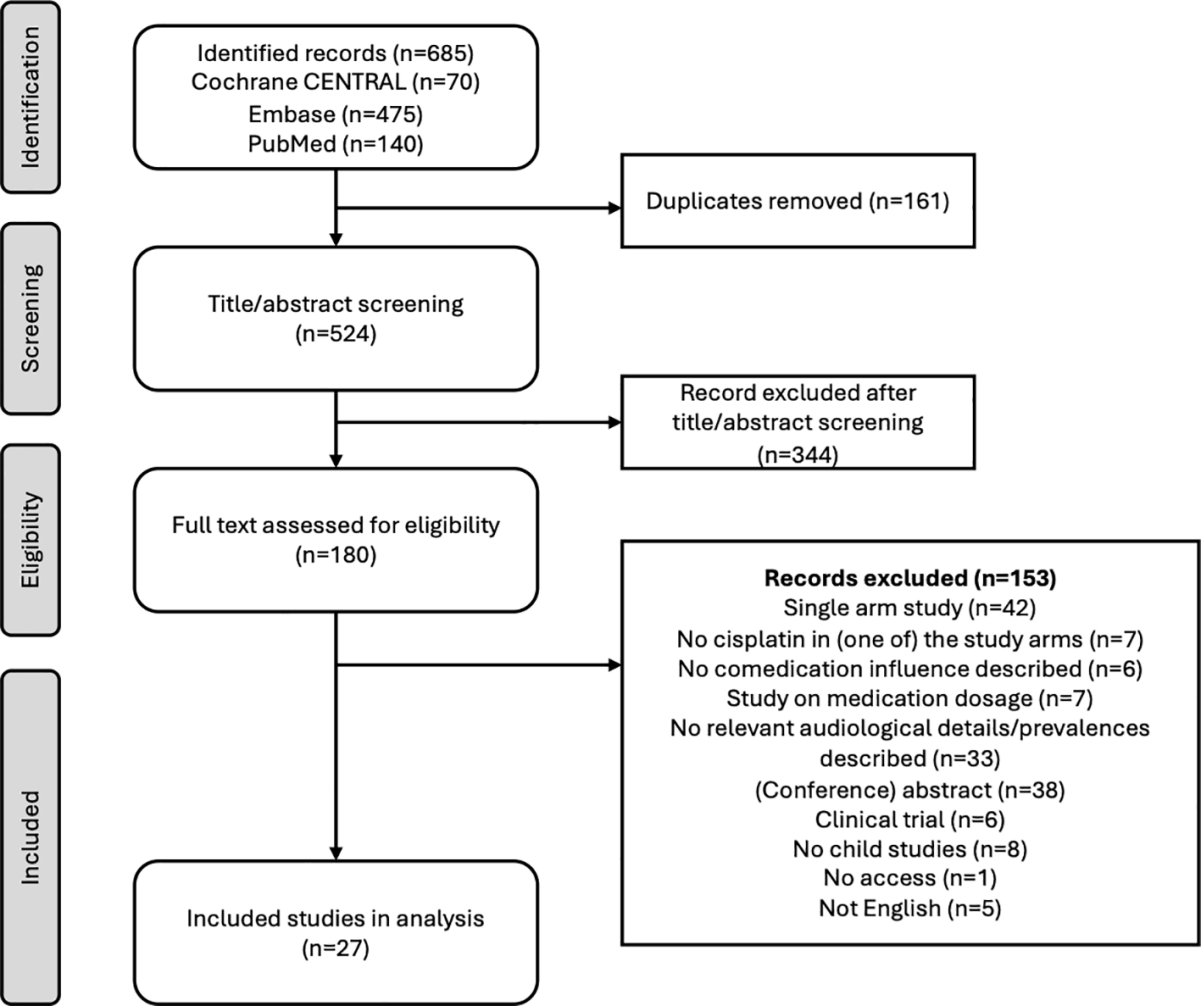
Flowchart of inclusion of studies in our narrative review search.

The literature search identified a total of 685 publications. After removal of duplicates, 526 studies were identified for title and abstract screening (**Figure 1**). After full text screening, 27 studies on the association of comedication with occurrence of CIHL were included in this review. The studies were published between 1990 and 2025 and included 28–1481 participants with evaluable audiograms (in total n=7007). In total, 17 studies assessed the association between chemotherapeutic agents and CIHL, five investigated loop diuretics, and 18 examined the effect of aminoglycosides and/or glycopeptides on CIHL (**Table 1**). Study designs consisted of 20 retrospective studies, four prospective studies, and three randomized control trials (RCTs). The distribution between male and female patients was similar in all studies.

**Table 1:** Pediatric cancer studies on the effect of comedication on cisplatin-induced hearing loss.

Study characteristics				Cohort characterizations			Comedication details			Hearing loss			Outcome		Risk of bias
Author and year of publication	Study design	Country – ISO code	Cohort and No. patients (n)	Disease studied	Age mean/median (range) [years] Cases/ controls	Male (%) Cases/ controls	Name of co- medication	Cumulative dosage comedication (mg/m^2^)	Cumulative dosage cisplatin, median (range) (mg/m^2^) Cases/ controls	Type of hearing loss assessment		Hearing loss classification system	Type OR	OR (95% CI)	(QUIPS overall)^1^
										≤ 3 years	> 3 years				
Chemotherapeutic agents															
*Vincristine*															
Diepstraten, 2024 (12)	Retro. study	NLD	National study (n=199)	Pediatric solid tumors	Median: 4 (0-18)	53	VCR	8.9 (1.5-17.7)^**^	467.4 (159.4-635.5)^**^	DPOAE, TEOAE, BERA, VRA	DPOAE, TEOAE, CPA, PTA (≥ 5 y)	Muenster (grade ≥2b)	Unadjusted OR	1.85 (1.03-3.30)	Low
												SIOP (grade ≥2)	Unadjusted OR	2.60 (1.45-4.69)	
Siemens, 2023 (36)	Retro. study	NLD, CAN	Multicenter study (n=371)	Pediatric cancer (cisplatin-treated)	Median: 6 (0-19)	54	VCR	NA	400 (100-810)^**^	PTA	PTA	CTCAE v5 (grade ≥2)	Crude OR	5.96 (3.56-10.00)	Low
Strebel, 2023 (41)	Retro. study	CHE	National cohort (n=270)	Pediatric cancer (cisplatin-treated)	Median: 7 (IQR: 2-12)	57	VCR	23 (IQR: 10.1-40.9)	NA	OAE, ABR	PTA, OAE, ABR	SIOP (grade ≥2)	Unadjusted OR	3.50 (2.00-6.00)	Moderate
Meijer, 2022 (42)	Retro. study	NLD, CAN	Multicenter study (n=368)	Pediatric cancer (cisplatin-treated)	Median: 6 (29 days - 19 years)	52	VCR	NA	400 (100-800)	OAE	PTA, OAE	SIOP (grade ≥2)	Unadjusted HR*	2.89 (2.08-4.02)	Low
Moke, 2021 (43)	Retro. study	USA, CAN	Multicenter study (n=1481)	Pediatric cancer (cisplatin-treated)	< 5y: n: 644 5 to < 15: n: 630 ≥ 15 y: n: 206	56	VCR	NA	Mean: 410 (50-1600)	ABR	PTA^‡^, ABR	SIOP (grade ≥2)	Unadjusted OR	5.77 (4.34-7.71)	Low
Vos, 2016 (32)	Retro. study	NLD	Multicenter study (n=156)	Pediatric osteosarcoma	Median: 14 (3-40)/14 (5-42)	54	VCR	NA	480 (140-720)^**^	–	CPA, PTA	Chang (grade >0)	Crude OR	3.85 (0.77-19.15)	Moderate
Castelán-Martínez, 2014 (38)	Retro. study	MEX	Single center study (n=59)	Pediatric cancer (cisplatin-treated)	Median: 11 (3-17)	53	VCR	NA	370 (170-695) >400^**^	PTA	PTA	CTCAE v4.03 (grade ≥1)	Unadjusted OR	1.70 (0.38-7.58)	Moderate
Hagleitner, 2015 (44)	Retro. study	NLD, ESP	Two center study (n=148)	Pediatric osteosarcoma	Median: NLD: 15 (5-40) ESP: 14 (4-29)	51	VCR	NA	500 (100-870)	–	CPA, PTA	CTCAE v3 (grade ≥2) SIOP (grade ≥2)	Crude OR	1.32 (0.38-4.54)	Moderate
Pussegoda, 2013 (35)	Retro. study	CAN	Multicenter study (n=317)	Pediatric cancer (cisplatin-treated)	Median: 6 (0-25)/ 10 (0-19)	59/ 50	VCR	NA	Cases: 400 (92-800)^**^ Controls: 400 (20-768)	NA	NA	CTCAE v3 (grade ≥2)	Crude OR	4.53 (2.73-7.50)	Moderate
Ross, 2009 (33)	Retro. study	USA	Multicenter study (n=162)	Pediatric cancer (cisplatin-treated)	Median: 6 (0-16)/ 9 (0-19)	61	VCR	NA	480 (120-720)^**^	NA	NA	CTCAE v3 (grade ≥2)	Crude OR	11.01 (0.63-192.8)	Moderate
*Other chemotherapeutic agents*															
Metwally, 2025 (39)	Retro. and pros. study arm	EGY	Single center study (n=168)	Pediatric standard-risk medulloblastoma	Median: 8 (6-11)	66	CPM (COG-A9961 vs COG-ACNS0331)	6000	Without CPM: 525 (450-595) With CPM: 413 (352-450)	NA	NA	CTCAE v5.0 (grade ≥3)	Crude OR	0.12 (0.03-0.51)	Moderate
Becktell, 2024 (45)	Retro. study	USA, CAN	Multicenter study (n=1257)	Pediatric osteosarcoma	Median: 14 (2-21)	52	CPM (MABCDP vs MAP)	BLM: 118 (0-371) CPM: 7280 (20-42010)	466 (60-4668)	NA	NA	CTCAE v4.03 (grade ≥3)	Crude OR	0.62 (0.38-1.02)	Moderate
Moore, 2023 (47)	Retro. study	USA	Single center study (n=66)	Pediatric cancer (NFS)	(10 months – 19 years)	NA	MTX	(60-144,000)	(120-600)	DPOAE, ABR	DPOAE, PTA	SIOP (grade ≥1)	Crude OR	1 (0.07-13.87)	Moderate
Castelán-Martínez, 2014 (38)	Retro. study	MEX	Single center study (n=59)	Pediatric cancer (cisplatin-treated)	Median: 11 (3-17)	53	DOX	NA	370 (170-695) >400^**^	PTA	PTA	CTCAE v4.03 (grade ≥1)	Unadjusted OR	0.46 (0.15-1.46)	Moderate
Perilongo, 2009 (48)	RCT	GBR	Single center study (n=255)	Pediatric standard-risk hepatoblastoma	Median: 14 months (0-134)	61	DOX	NA	NA	NA	NA	Brock (grade ≥2)	Crude OR	0.92 (0.43-1.98)	Low
Packer, 2006 (46)	RCT	USA	Multicenter study (n=379)	Pediatric average-risk medullablastoma	3-4y: n: 65 5-9y: n: 193 10-14y: n: 96 15-19y: n: 25	59	CPM vs CCNU	CPM: 16,000 CCNU: 600	600	NA	NA	(grade ≥3)	Crude OR	0.77 (0.49-1.23)	Moderate
Winkler, 1990 (40)	RCT	AUT, CHE	Two center study (n=65)	Pediatric osteosarcoma	<15y: 49 >15y: 60 Median: 15.6 (2-40)	56	IFO vs BLM (COSS-80 vs COSS-86)	IFO: 6000 BLM: 12	Cisplatin (IFO): (180-240) Cisplatin (BLM): 120	NA	NA	NA	Crude OR	19.86 (2.45-161.3)	Moderate
Loop diuretics															
Diepstraten, 2024 (12)	Retro. study	NLD	National study (n=199)	Pediatric solid tumors	Median: 4 (0-18)	53	Furo	NA	467.4 (159.4-635.5)^**^	DPOAE, TEOAE, BERA, VRA	DPOAE, TEOAE, CPA, PTA (≥ 5 y)	Muenster (grade ≥2b)	Unadjusted OR	1.54 (0.85-2.77)	Low
												SIOP (grade ≥2)	Unadjusted OR	1.30 (0.72-2.36)	
Siemens, 2023 (36)	Retro. study	NLD, CAN	Multicenter study (n=371)	Pediatric cancer (cisplatin-treated)	Median: 6 (0-19)	54	Furo	NA	400 (100-810)^**^	PTA	PTA	CTCAE v5 (grade ≥2)	Crude OR	2.92 (0.14-61.26)	Low
Meijer, 2022 (42)	Retro. study	NLD, CAN	Multicenter study (n=368)	Pediatric cancer (cisplatin-treated)	Median: 6 (29 days – 19 years)	52	Furo	NA	400 (100-810)	PTA, OAE	PTA, OAE	SIOP (grade ≥2)	Unadjusted OR	1.26 (0.93-1.72)	Low
Clemens, 2016 (27)	Retro. study	NLD	Multicenter (n=451)	Pediatric cancer (platinum-treated)	Median: 5 (0.0-19)	50	Furo	NA	480 (76-990)^**^	NA	PTA	Muenste (grade ≥2b)	Unadjusted OR	2.30 (1.40-3.90)	Low
Olgun, 2016 (31)	Pros. study	TUR	Two center study (n=72)	Pediatric cancer (cisplatin-treated)	Mean: 10 (1-17)	48	Furo	NA	>400^**^	DPOAE, ABR	DPOAE, PTA	Brock (grade ≥2)	Crude OR	4.60 (0.54-39.1)	Moderate
												Muenster (grade ≥2)	Crude OR	2.80 (0.54-14.6)	
Antibiotics (aminoglycosides and glycopeptides)															
Diepstraten, 2024 (12)	Retro. study	NLD	National study (n=199)	Pediatric solid tumors	Median: 4 (0-18)	53	b) Genta d) Vanco e) Teico	NA	467.4 (159.4-635.5)^**^	DPOAE, TEOAE, BERA, VRA	DPOAE, TEOAE, CPA, PTA (≥ 5 y)	Muenster (grade ≥2b)	Unadjusted OR	b) 2.28 (1.08-4.78) d) 3.09 (1.71-5.60) e) 1.66 (0.82-3.36)	Low
												SIOP (grade ≥2)	Unadjusted OR	b) 2.17 (1.10-4.31) d) 2.71 (1.49-4.92) e) 1.55 (0.80-3.01)	
Romano, 2023 (28)	Retro. study	ITA	Single center study (n=53)	Pediatric cancer (platinum-treated)	Median: 7 (SD: 4.5)	57	Not specified	7286 (SD: 10813.7)^**^	417.9 (SD: 236.5)^**^	VRA (<30 m)	CPA (≥ 30 m) PTA (≥ 5 y)	SIOP (grade >0)	Unadjusted HR*	1.19 (0.42-1.96)	Moderate
Siemens, 2023 (36)	Retro. study	NLD, CAN	Multicenter study (n=371)	Pediatric cancer (cisplatin-treated)	Median: 6 (0-19)	54	b) Genta c) Tobra	NA	400 (100-810)^**^	PTA	PTA	CTCAE v5 (grade ≥2)	Crude OR	b) 2.28 (0.25-20.64) c) 2.20 (0.71-6.76)	Low
Sherief, 2022 (37)	Pros. study	EGY	Two center study (n=64)	Pediatric solid tumors	Mean: 13 (SD 5.6)/8 (SD 5.4)	63	a) Amika	NA	Mean 851 SD 602^**^	PTA	PTA	Brock (grade ≥2)	Crude OR	a) 0.30 (0.02-5.88)	Moderate
Sriyapai, 2022 (29)	Retro. study	THA	Single center study (n=47)	Pediatric solid tumors	Median: 7 (1.3-14.8)	49	Not specified	NA	423.1 (118-1080) >400^**^	ASSR, audiogram NA	ASSR, audiogram NA	CTCAE v5 (grade ≥2)	Crude OR	13.3 (0.69-255.0)	Moderate
Meijer, 2022 (42)	Retro. study	NLD, CAN	Multicenter study (n=368)	Pediatric cancer (cisplatin-treated)	Median: 6 (29 days – 19 years)	52	NS*^†^*	Median [range] per 5-d increase: Genta 6 [1-34]^**^ Tobra 12.5 [1-73]^**^ Vanco 11 [1-52]^**^	400 (100-810)	PTA, OAE	PTA, OAE	SIOP (grade ≥2)	Unadjusted HR*	1.90 (1.27-3.01)	Low
Olgun, 2021 (30)	Retro. study	TUR	Single center study (n=84)	Pediatric cancer head and neck region	Mean: 88 months (1-202)	58	Not specified	NA	NA	DPOAE, ABR	DPOAE, PTA	Brock (grade ≥2)	Crude OR	1.51 (0.54-4.25)	Moderate
												Chang (grade ≥2)	Crude OR	1.11 (0.40-3.08)	
										Hearing loss classification system	Type OR	Muenster (grade ≥2b)	Crude OR	1.29 (0.47-3.60)	
Turan, 2019 (49)	Pros. study	TUR	Single center study (n=50)	Pediatric cancer (cisplatin-treated)	Mean: 9 (SD 5.2)/10 (SD 5.2)	42	Not specified	NA	Mean: 319 SD 244/ 241.1 SD: 107.6	ABR, DPOAE	ABR, DPOAE, PTA	Brock, NCI, Chang	Crude OR	0.47 (0.12-1.80)	Moderate
Clemens, 2016 (27)	Retro. study	NLD	Multicenter study (n=451)	Pediatric cancer (platinum-treated)	Median: 5 (0-19)	50	b) Genta c) Tobra d) Vanco	NA	480 (76-990)^**^	Not performed	PTA	Muenster (grade ≥2b)	Unadjusted OR	b) 1.30 (0.80-2.40) c) 1.10 (0.70-1.80) d) 1.30 (0.90-1.90)	Low
Olgun, 2016 (31)	Pros. study	TUR	Single center study (n=72)	Pediatric cancer (cisplatin-treated)	Mean: 10 (1-17)	48	Not specified	NA	>400^**^	DPOAE, ABR	DPOAE, PTA	Brock (grade ≥2)	Unadjusted OR	3.84 (1.18-12.47)	Moderate
												Muenster (grade ≥2)	Unadjusted OR	3.55 (1.18-10.66)	
Castelán-Martínez, 2014 (38)	Retro. study	MEX	Single center study (n=59)	Pediatric cancer (cisplatin-treated)	Median: 11 (3-17)	53	a) Amika	NA	370 (170-695) >400^**^	PTA	PTA	CTCAE v4.03 (grade ≥1)	Unadjusted OR	a) 0.77 (0.14-4.15)	Moderate
Hagleitner, 2015 (44)	Retro. study	NLD, ESP	Two center study (n=148)	Pediatric osteosarcoma	Median: NLD: 15 (5-40) EPS: 14 (4-29)	51	Not specified	NA	500 (100-870)	–	CPA, PTA	CTCAE v3 (grade ≥2)	Crude OR	2.79 (1.16-6.74)	Moderate
												SIOP (grade ≥2)	Crude OR	2.79 (1.16-6.74)	
Landier, 2014 (50)	Retro. study	COG	Multicenter study (n=333)	Pediatric high-risk neuroblastoma	Median: 3 (0-29)	56	Not specified^††^	NA	400	–	PTA	Brock (grade ≥3)	Adjusted OR	5.1 (1.7-14.9)	Low
												Chang(grade≥2b)	Adjusted OR	2.2 (1.2-4.3)^†††^	
												CTCAE v3 (grade ≥3)	Adjusted OR	1.8 (0.86-3.7)	
Choeyprasert, 2013 (34)	Retro. study	THA	Single center study (n=68)	Pediatric solid tumors	Mean: 8 (4-9)	40	Not specified	NA	600 (170-1050) ^**^	NA	CPA, PTA	Brock (grade ≥1)	Crude OR	0.48 (0.14-1.62)	Moderate
Pussegoda, 2013 (35)	Retro. study	CAN	Multicenter study (n=317)	Pediatric cancer (cisplatin-treated)	Median: 6 (0-25)/10 (0-19)	59/ 50	b) Genta c) Tobra d) Vanco	NA	400 (92-800)/ 400 (20-768)	NA	NA	CTCAE v3 (grade ≥2)	Crude OR	b) 1.05 (0.60-1.82) c) 1.25 (0.75-2.09) d) 1.42 (0.83-2.45)	Moderate
Lewis, 2009 (51)	Retro. study	USA	Single center study (n=36)	Pediatric osteosarcoma	Median: 14 (3-18)	39	Not specified d) Vanco	NA	360 (210-480)	–	CPA, “hand-raising”	Brock (grade ≥1)	Crude OR	2.29 (0.59-8.91) d) 2.29 (0.59-8.91)	Moderate
Ross, 2009 (33)	Retro. study	CAN	National study (n=162)	Pediatric cancer (cisplatin-treated)	Median: 6 (0-16)/9 (0-19)	61	b) Genta c) Tobra d) Vanco	NA	480 (120-720)^**^	NA	NA	CTCAE v3 (grade ≥2)	Crude OR	b) 1.84 (0.49-6.98) c) 0.94 (0.40-2.20) d) 1.16 (0.42-3.25)	Moderate
Kretschmar, 1990 (52)	Pros. study	USA	Single center study (n=28)	Pediatric brain tumors	Median: 5 (5m-25y)	NA	Not specified	NA	300	ABR, VRA (<30 m)	ABR, CPA(≥30 m), PTA (≥5 y)	NA	Crude OR	0.57 (0.05-6.08)	Moderate

^1^The overall risk of bias using the QUIPS tool was primarily based on study participation, prognostic factor measurement, and study confounding, as these domains are most critical for assessing the validity of the association between medication exposure and hearing loss. The complete assessment is listed in **Table 2**.

*HR: hazard ratio instead of odds ratio; ^**^total cumulative dose associated with HL (not mean/median of whole cohort); ^‡^Assumed audiological assessment based on in-text details.

^†^Aminoglycosides (gentamycin, tobramycin and/or vancomycin) administered >30 days, type not specified;^††^Hospitalization for infection as surrogate for aminoglycoside treatment;^†††^The OR is read from the graph.

ABR, auditory brainstem response; Amika, amikacin; ASSR, auditory steady-state response; BERA, brainstem evoked response audiometry; BLM, bleomycin; CCNU, lomustine; CI, confidence interval; COG, children’s oncology group; COSS, cooperative osteosarcoma study; CPA, conditioned play audiometry; CTC, Common Toxicity Criteria; CTCAE, Common terminology criteria for adverse events; CPM, cyclophosphamide; DOX, doxorubicin; DPOAE, distortion product OAE; Genta, gentamycin; HR, hazard ratio; IFO, ifosfamide; IQR, interquartile range; m, months; MAP, methotrexate + doxorubicin + cisplatin; MABCDP, MAP + bleomycin + dactinomycin + cyclophosphamide; MTX, methotrexate; NA, not available/not specified; NFS, not further specified; OAE, otoacoustic emissions; OR, odds ratio; Pros., prospective; PTA, pure tone audiometry; Retro., retrospective; RCT, randomized control trial; SIOP, International Society of Paediatric Oncology; Teico, teicoplanin; TEOAE, transitory evoked OAE; Tobra, tobramycin; SD, standard deviation; v, version; Vanco, vancomycin; VRA, visual reinforcement audiometry; y, years.

A meta-analysis was performed to assess the risk of developing CIHL with comedication of vincristine, cyclophosphamide, furosemide, and antibiotics. The funnel plots were roughly symmetrical, except for cyclophosphamide (**Supplementary Figure 1**), suggesting a small likelihood of publication bias. The exact prevalences and possible confounding factors are listed in **Supplementary Table 2**. Overall, most studies showed moderate risk of bias, mainly due to limitations in confounding control and variability in hearing loss measurements and definitions (**Table 2**).

**Table 2:** Risk of bias assessment using the QUIPS tool.

Study	Study participation	Study attrition	Prognostic factor measurement	Outcome measurement	Study confounding	Statistical analysis & reporting	Overall^1^
Metwally, (39)	Low	Low	Low	Moderate	High	Moderate	Moderate
Becktell, (45)	Low	Moderate	Low	Moderate	High	Moderate	Moderate
Diepstraten, (12)	Low	Low	Low	Low	High	Low	Low
Moore, (47)	Moderate	Moderate	Moderate	Moderate	High	Moderate	Moderate
Romano, (28)	Moderate	Moderate	Low	Low	Moderate	Moderate	Moderate
Siemens, (36)	Low	Low	Low	Low	High	Moderate	Low
Strebel, (41)	Low	Moderate	Moderate	Low	High	Low	Moderate
Meijer, (42)	Low	Low	Low	Low	High	Low	Low
Sherief, (37)	Moderate	Low	Low	Low	High	Moderate	Moderate
Sriyapai, (29)	Moderate	Low	Low	Moderate	High	Moderate	Moderate
Moke, (43)	Low	Low	Low	Low	Low	Low	Low
Olgun, (30)	Moderate	Low	Low	Low	High	Moderate	Moderate
Turan, (49)	Moderate	Low	Moderate	Low	High	Moderate	Moderate
Clemens, (27)	Low	Moderate	Low	Low	Moderate	Low	Low
Olgun, (31)	Moderate	Low	Moderate	Low	High	Moderate	Moderate
Vos, (32)	Low	Moderate	Low	Low	High	Moderate	Moderate
Hagleitner, (44)	Low	Low	Low	Low	High	Moderate	Moderate
Castelán-Martínez, (38)	Moderate	Low	Low	Low	Moderate	Moderate	Moderate
Landier, (50)	Low	Moderate	Low	Low	Moderate	Low	Low
Choeyprasert, (34)	Moderate	Moderate	Low	Low	High	Moderate	Moderate
Pussegoda, (35)	Low	Low	Low	Moderate	High	Moderate	Moderate
Perilongo, (48)	Low	Low	Low	Moderate	Low	Low	Low
Lewis, (51)	Moderate	Low	Low	Moderate	Moderate	Moderate	Moderate
Ross, (33)	Low	Moderate	Low	Moderate	High	Moderate	Moderate
Packer, (46)	Low	Low	Moderate	Moderate	Moderate	Moderate	Moderate
Kretschmar, (52)	Moderate	High	Moderate	Moderate	High	Moderate	Moderate
Winkler, (40)	Moderate	Moderate	Moderate	Moderate	Moderate	Moderate	Moderate

^1^The overall risk of bias was primarily based on study participation, prognostic factor measurement, and study confounding, as these domains are most critical for assessing the validity of the association between medication exposure and hearing loss. Color coding was used to indicate the risk of bias using the QUIPS tool: green = low risk of bias, yellow = moderate risk of bias, and red = high risk of bias.

### 3.1 Cisplatin dose

The reported median or mean cumulative cisplatin dose ranged between 281 and 821 mg/m^2^ (**Table 1**). All studies that found a significant association between cumulative cisplatin dose and hearing loss involved doses of 400 mg/m^2^ or higher (12, 27–38). Three studies used different cisplatin doses between the two study arms investigating the association with comedication (35, 39, 40). In all studies, the study arm with the highest cisplatin dose showed the highest OR for developing CIHL regardless of the additional comedication.

### 3.2 Chemotherapeutic agents in association with CIHL risk

Studies investigating the effect of additional chemotherapeutic agents on CIHL were included in this review (**Table 1**). Vincristine was the most frequently investigated comedication for CIHL (n=10 studies, n=3531) (12, 32, 33, 35, 36, 38, 41–44). The studies generally had large sample sizes with an approximately equal male-to-female ratio. The median cumulative cisplatin dose was ≥ 400 mg/m^2^ in all but one study, where it was 370 mg/m^2^ (38). The risk-related vincristine dose was absent in eight of the ten studies, while the type of age-appropriate audiological assessments was described in eight of the ten. The CTCAE classification system was most frequently employed to grade hearing outcomes (n=5; three used version 3, one used version 4.03 and one used version 5), followed by the SIOP system (n=4, concordance with CTCAE: κ=0.797) (23)). One study applied the Chang classification (concordance with CTCAE κ=0.681; with SIOP κ=0.845) (23)). All ORs pointed in the same direction, with six out of ten studies showing a significantly increased risk of CIHL.

**Figure 2:**
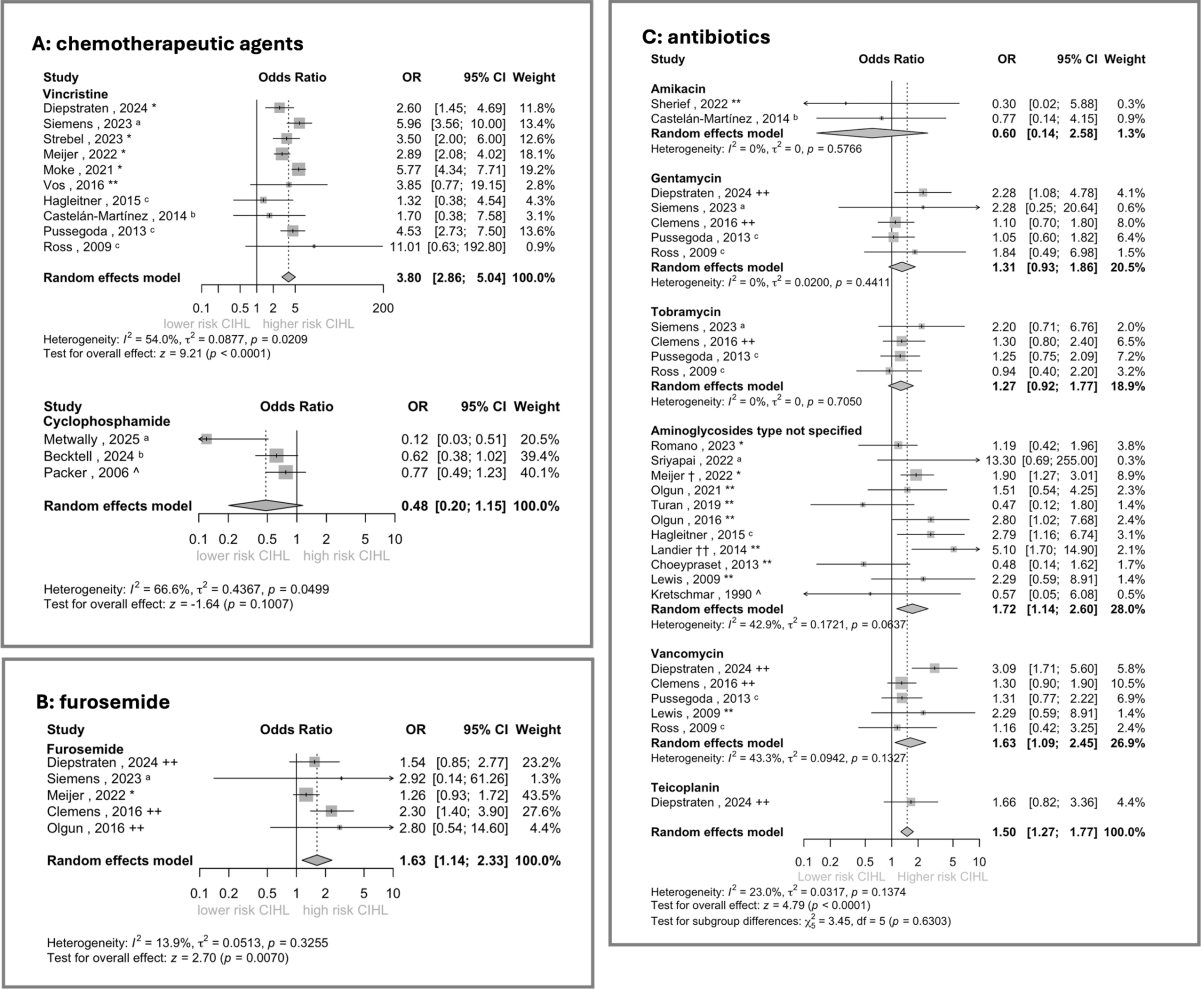
The meta-analyses of the risk of developing CIHL by chemotherapeutic agents **(A)**, furosemide **(B)**, and antibiotics **(C)** as comedication. Classification: CTCAE. Versions identified as follows: ^a^Version 5, ^b^Version 4.03, ^c^Version 3. Other classifications: *SIOP, **Brock, ^+^Chang, ^++^ Muenster, ^Classification system not specified. ^†^Aminoglycosides (gentamycin, tobramycin and/or vancomycin) administered >30 days, type not specified; ^††^Hospitalization for infection as surrogate for aminoglycoside treatment. Squares (▪) represent the ORs for individual studies, with square size proportional to the study weight in the meta-analysis; horizontal lines indicate 95% CIs. Diamonds (♦) represent the pooled effect estimates for each subgroup and for all cohorts combined. The solid line (—) represents OR = 1 and the dashed line (-–) shows the OR of the overall meta-analysis. CI, confidence interval; OR, odds ratio.

The meta-analysis demonstrated moderate heterogeneity (I^2^ = 54%, τ^2^ = 0.09, Q-test p=0.02), indicating inter-study variability in CIHL risk (**Figure 2A**). Nevertheless, the random-effects model revealed a significant increased risk of CIHL with vincristine cotreatment (OR: 3.80, 95% CI: 2.86-5.04).

The impact of other chemotherapeutic agents on CIHL has been less frequently studied (n=7 studies, n=2249 patients) (38–40, 45–48). No studies on vinblastine, etoposide, or 5-fluorouracil were identified from the literature search. This review included three studies on cyclophosphamide, two on doxorubicin, and single studies on methotrexate and ifosfamide. Study designs varied, including four single-center, one two-center, and two multicenter studies. Reported cumulative cisplatin doses varied widely (range 60–4668 mg/m^2^). The high dose of 4668 mg/m^2^ reported by Becktell et al. was an extreme outlier compared with the other reported ranges (45). The grading systems varied, CTCAE was used in three studies (two used version 4.03 and one used version 5), Brock and SIOP once each, and two studies did not specify a system. Most studies reported CIHL prevalence rather than unadjusted ORs (6 of 7 studies).

Findings also differed by agent: cyclophosphamide and doxorubicin showed a trend opposite toward an increased CIHL risk, methotrexate showed no effect, and ifosfamide demonstrated an association with increased CIHL risk. No meta-analyses of the studies on doxorubicin, methotrexate, and ifosfamide were performed due to the limited number of studies. The meta-analysis of the three studies on the effect cyclophosphamide studies showed a nonsignificant association opposite to an increased risk of CIHL (OR: 0.48, 95% CI: 0.20-1.15) (**Figure 2A**), with substantial heterogeneity between the studies (I^2^ = 67%, τ^2^ = 0.44, Q-test p=0.05).

### 3.3 Diuretics in association with CIHL risk

Five different retrospective studies (n=1461 patients) investigating loop diuretics and CIHL were included in this review (**Table 1**) (12, 27, 31, 36, 42). All studies examined furosemide, none of which reported cumulative doses. Three of the included studies applied the Muenster classification, one the CTCAE v5 system, and one the SIOP system (concordance with Muenster κ=0.857 and CTCAE κ=0.744, respectively) (23). All ORs were into the same direction, suggesting an increased risk of CIHL.

The random-effects model showed limited heterogeneity (I^2^ = 14%, τ^2^ = 0.05, Q-test p=0.33) and demonstrated a significant increased risk of hearing loss associated with furosemide use (OR: 1.63, 95% CI: 1.14-2.33) (**Figure 2B**).

### 3.4 Antibiotics in association with CIHL risk

The potential increased risk of CIHL associated with antibiotics was investigated in 18 studies including 2910 patients (12, 27–31, 33–38, 42, 44, 49–52). These studies examined aminoglycosides and/or glycopeptides, including vancomycin, amikacin, gentamicin, tobramycin, or unspecified aminoglycosides.

Our review included 14 retrospective studies and four prospective studies on aminoglycosides and/or glycopeptides (**Table 1**). The type of studies were mostly single center studies (n=9), followed by five multicenter studies, two national cohort studies, and two two-center studies. Median age of patients ranged from 3.3 to 13 years.

Two studies evaluated the association between amikacin and CIHL (37, 38). Hearing loss was evaluated using pure-tone audiometry (PTA) and graded utilizing the Brock and CTCAE v4.03 classifications. The ORs were in the opposite direction of increased risk of CIHL, contradictory to the other investigated aminoglycosides and glycopeptides. The effect was not significant with large confidence intervals (OR: 0.60, 95% CI: 0.14-2.58).

Five studies assessed gentamycin and four studies assessed tobramycin (12, 27, 33, 35, 36). The median age of patients with CIHL in the studies was between 4 and 6 years. In all studies, cumulative cisplatin dose was ≥ 400 mg/m^2^. For both aminoglycosides, CTCAE was used in three studies (twice v3 and once v5); the Muenster scale was applied in one study for tobramycin and in two studies for gentamycin. All ORs for gentamycin indicated an increased risk of CIHL, whereas for tobramycin, one OR was suggested in the opposite direction with a wide range. The meta-analysis of aminoglycoside gentamycin showed a trend towards an increased risk of developing CIHL, although the effect was not statistically significant and neither was the effect for tobramycin (OR: 1.31, 95% CI: 0.93-1.86 and OR: 1.27, 95% CI: 0.92-1.77, respectively).

For the studies (n=11) that did not specify the type of aminoglycoside, eight ORs suggested an increased CIHL risk and three showed the opposite direction (28, 29, 31, 34, 42, 44, 50–52). Brock classification was utilized in six studies, SIOP in two, CTCAE in two (v3 and v5), and in one study it was not specified. Two studies specified the total cumulative aminoglycoside dose: Romano et al. reported 7286 mg/m^2^ for aminoglycosides (unspecified) and Meijer et al. reported 6, 12.5 and 11 mg/m^2^ for gentamycin, tobramycin and vancomycin per 5-day increase, respectively (28, 42). The meta-analysis of the unspecified aminoglycosides showed a significant increased risk of CIHL (OR: 1.72, 95% CI: 1.14-2.60).

Five studies investigated the glycopeptide vancomycin, all showing an OR into the direction of increased CIHL risk (12, 27, 33, 35, 51). Median cumulative cisplatin dose was ≥ 400 mg/m^2^ in four studies and not specified in one. The studies classified hearing loss using CTCAE v3 (n=2), Muenster (n=2) and Brock (n=1). Heterogeneity between studies was limited (I^2^ = 43%, τ^2^ = 0.09, p=0.13), and the increased risk was significant (OR: 1.63, 95% CI: 1.09-2.45). The findings for the single study investigating teicoplanin were not significant (12).

The overall meta-analysis of aminoglycoside and glycopeptide antibiotics showed an OR of 1.50 (95% CI: 1.27-1.77). The model showed limited heterogeneity between the studies overall (I^2^ = 23%, τ^2^ = 0.03, Q-test p=0.14) (**Figure 2C**).

## 4 Discussion

Cisplatin is a cornerstone chemotherapeutic agent in the treatment of various pediatric malignancies, but ototoxicity is a significant side effect. This is the first review to summarize studies on various comedications of cisplatin in relation to CIHL. Altogether 27 studies with a total of 7007 patients were identified. Vincristine, furosemide, aminoglycosides and vancomycin were associated with an increased risk of CIHL in the random-effects models of the meta-analyses. The results remained statistically significant when analyzed using a fixed-effects model, indicating the robustness of the findings. In the literature, some small studies and case reports suggest that these comedications may have an independent ototoxic effect (**Supplementary Table 3**), and this review highlights their potential additive contribution to the development of CIHL. Despite the well-known effects on quality of life, socioeconomic consequences and physical functioning, no universal clinical standards currently exist for the management of treatment-related ototoxicity (53–55). We advocate for clear international guidelines on cancer-treatment related ototoxicity, including the effect of concomitant medication.

While vincristine has been suggestively associated with ototoxicity in individual case reports (21, 56, 57), its independent ototoxic potential remains uncertain, as prospective studies have not consistently confirmed this effect (20, 58, 59). Bokemeyer et al. (92) observed a rising incidence of hearing loss with increasing vincristine doses in adult patients receiving cisplatin, supporting the possibility of a dose-dependent interaction. Vincristine is primarily known for its neurotoxic side effects, including peripheral neuropathy (60–62) and, in rare cases, optic and acoustic neuropathy leading to blindness (63, 64) and deafness (65). However, this review shows that vincristine is associated with a statistically significantly increase of CIHL in combination with cisplatin, underscoring the need for further research into its interactive effects with cisplatin, particularly in pediatric oncology settings. The enhanced risk of CIHL observed with vincristine may reflect neurotoxic rather than cochlear damage. The available circumstantial evidence suggests that vincristine could increase cisplatin-induced damage to the auditory system, potentially via effects on the auditory nerve (43).

The remaining chemotherapeutic agents identified in the literature represented a limited number of studies, often with considerable heterogeneity. As a result, no significant association with CIHL was found for cyclophosphamide, doxorubicin, methotrexate, and ifosfamide. Treatment regimens including cyclophosphamide were not associated with an increased risk of CIHL, though cumulative cisplatin dose likely influenced outcomes. The cumulative cisplatin doses were higher in the comparable study arms without cyclophosphamide, which had the highest CIHL risk (39, 45). Another study with similar cisplatin doses of 600 mg/m^2^ in both arms found no increased risk (46). These findings suggest cisplatin dose differences may contribute to variability in effect of cyclophosphamide on CIHL. New RCTs or prospective studies could identify any potential effects of these chemotherapeutics.

The literature search yielded no studies investigating the impact of other chemotherapeutic agents, included in the search terms, on CIHL. Carboplatin is not included in this narrative review of comedication associated with CIHL. Its role and associated risk in CIHL are well established (55, 66–68), and it is often substituted for cisplatin during therapy in case of early CIHL. We instead focused on other comedication that may increase CIHL risk.

This review suggests that furosemide significantly increases the risk of CIHL. This effect may be attributable to the action of loop diuretics on tight junctions in the blood-cochlea barrier, thereby facilitating the entry of toxins and promoting cochlear injury (18, 19). The fact that furosemide is frequently prescribed in patients receiving higher doses of cisplatin to manage fluid retention may explain the association between this comedication and hearing loss. This would explain the non-significant findings in four of five studies in the meta-analysis (12, 31, 36, 42), in combination with low heterogeneity in our meta-analysis.

The pooled result of the meta-analysis assessing the risk on CIHL by aminoglycosides and glycopeptides showed an increased risk. These findings are in line with established mechanisms of cochlear injury mediated through oxidative stress, mitochondrial dysfunction, and disruption of cochlear homeostasis (69–71). Studies specifying aminoglycoside types showed no significant CIHL risk, while the meta-analysis including unspecified aminoglycosides and overall data did. This may be due to the additive or synergistic effects of aminoglycosides and glycopeptides on each other. Brummett et al. found no increased risk of hearing loss due to vancomycin, yet it enhanced the risk of gentamicin-ototoxicity (72). In this review, however, concurrent vancomycin and cisplatin treatment was significantly associated with increased CIHL risk. Limited data on amikacin suggest a lower CIHL risk, conflicting with literature documenting its ototoxicity (73, 74). This may reflect a lack of pediatric studies evaluating the risk of CIHL associated with amikacin or the influence of prior antibiotic adjustments. Moreover, the World Health Organization advises against the combined use of amikacin and cisplatin (75, 76).

Furthermore, it remains uncertain whether the elevated risk associated with certain antibiotics reflects a true synergistic mechanism or merely an additive effect. Animal studies support gentamicin-cisplatin synergy (77, 78), but further research is needed to clarify these interactions in pediatric cancer patients treated with cisplatin (22). In particular, the pathophysiological interaction between aminoglycosides, glycopeptides and CIHL warrants further investigation. Currently, harm-reduction strategies addressing the combined treatment of cisplatin and comedication are lacking (71, 79, 80). Nevertheless, awareness of the need to minimize avoidable exposure to ototoxic antibiotics increased in pediatric oncology (81). While otoprotective strategies for CIHL are rising, these largely focus on the direct ototoxic effects of cisplatin itself (10, 11). Future efforts should therefore prioritize the development of standardized audiological surveillance guidelines, therapeutic drug monitoring and the investigation of less toxic antibiotic alternatives.

No studies were identified on the effect of antiviral, antifungal, or antiemetic drugs on the risk of CIHL. One patient receiving amphotericin B developed hearing loss (52), consistent with case reports linking this drug to ototoxicity (82). Though, its interaction with cisplatin has not been studied in pediatric oncology. The effects of dexamethasone and ondansetron in combination with cisplatin were assessed in one study, but no data on hearing loss outcomes were reported (83).

A challenge in interpreting the increased CIHL risk caused by comedication lies in the variation between the dose and timing of cisplatin as well as the other agents. There is heterogeneity among the included studies, and the lack of RCTs requires careful interpretation. Especially since the mechanistic pathways by which drugs amplify cisplatin ototoxicity are not yet fully understood (5, 84, 85). Additionally, while data extraction was independently verified, the selection of studies was not. Several confounding factors likely influenced the observed associations. To be able to compare the findings of different studies in a meta-analysis, the unadjusted ORs or crude ORs are used, which influences the results as the risk is not adjusted for confounding factors. **Supplementary Table 2** lists possible treatment-related confounding factors as suggested in the available articles. In polychemotherapy regimens, isolating the independent ototoxic contribution of a single agent is inherently difficult. In addition, radiotherapy, which was administered concurrently with cisplatin in multiple studies (12, 28–31, 33–36, 39, 41–43, 45–47, 49, 52, 86), is itself a known contributor to hearing loss and may have skewed the reported ORs (87–89). Additionally, vincristine is a radiosensitizer and may synergistically enhance ototoxicity when combined with radiotherapy (43). In many studies, comparison groups differed only by presence or absence of a single agent, but were not controlled for additional ototoxic agents, further limiting the interpretability of isolated drug effects.

Another important risk factor for CIHL is age, particularly in children under five years (6, 42), and as most studies did not stratify results by age, this factor is often not adjusted for. Future studies may investigate the specific influence of age on the effect of concomitant medication on cisplatin-induced hearing loss, as pharmacokinetics, pharmacodynamics, and baseline cochlear sensitivity differ across developmental stages. Rapid onset of ototoxicity in younger patients (aged 0–5 years) has previously been reported (42, 55). Although the underlying mechanism remains unclear, we hypothesize that higher infection rates in younger patients (90), and therefore more frequent exposure to concomitant medications, may contribute to this increased risk.

The heterogeneity in grading systems used to assess ototoxicity further complicates data synthesis, although high concordance between these systems has been demonstrated (23). CTCAE version 3 defines grades based on clinical impact while later versions establish grading based on decibel loss, which may further increase heterogeneity between studies. Additionally, variations in the thresholds and assessment methods used to define clinically significant hearing loss introduced further inconsistencies in reported outcomes. Moreover, baseline audiometry assessments were lacking in most studies, so pre-existing hearing loss and its cause cannot be determined.

Furthermore, this review confirms the cisplatin dose-related risk factor of developing hearing loss. In line with earlier research, a threshold of 400 mg/m^2^ cisplatin has been found to be associated with CIHL (17). Therefore, the outcomes of studies having different cisplatin doses between study arms should be interpreted more carefully (35, 39, 40). Both the total cumulative dose and the daily and cycle dose are known to be associated with ototoxicity, with doses below 60 mg/m^2^ being less ototoxic than higher doses (91). Differences in the cycle and daily doses of cisplatin may have contributed to the variability in outcomes between two studies (40, 51). In addition, several studies reported real-time dose adjustments to cisplatin following early signs of ototoxicity (27, 28, 32, 33, 35, 36, 40, 41, 43, 44, 50). While this is a clinically appropriate intervention, it may have led to an underestimation of the cumulative ototoxic burden associated with specific comedication. Moke et al. (43) did not find an association between dose reduction and differences in outcomes.

Despite current limitations, this review provides the first comprehensive overview linking comedication to an increased risk of CIHL in pediatric cancer patients. Our findings highlight the multifactorial etiology of CIHL and underscore the need for heightened clinical awareness when prescribing adjunct therapies. Vincristine, furosemide, ahminoglycosides, and vancomycin are associated with the risk of CIHL in pediatric cancer patients. Future prospective studies employing standardized outcome measures and rigorous control of confounders are essential to elucidate drug interactions, clarify causal mechanisms, and inform universal guidelines for comedication management and audiological monitoring.
