# Supplementary material for: Comedication that increase the risk of cisplatin-induced hearing loss in pediatric cancer patients: a narrative literature review and meta-analysis
Source: Front Oncol. 2026 May 13;16:1769234. doi: 10.3389/fonc.2026.1769234 (PMC13212217; doi:10.3389/fonc.2026.1769234)
Supplement: Supplementary file 1 [file DataSheet1.pdf]

Supplementary materials: Comedication that Increase the Risk of Cisplatin-Induced Hearing Loss in Pediatric Cancer Patients: *A Narrative Literature Review and Meta-Analysis*

Supplementary Table 1: literature search terms

**Cochrane CENTRAL**
(vincristine OR vinblastine OR cyclophosphamide OR ifosfamide OR etoposide OR doxorubicin OR fluorouracil OR "5-fluorouracil" OR "5FU" OR "5-FU" OR "5 fluorouracil" OR adrucil OR HD-MTX OR methotrexate OR lomustine OR aminoglyco* OR glycopep* OR vancomycin OR teicoplanin OR tobramycin OR gentamycin* OR amikacin OR diuretic* OR furosemide OR lasix OR spironolactone OR aldactone OR hydrochlorothiazide OR microzide OR bumetanide OR bumex OR burinex OR antifungal OR amphotericin* OR fungizone OR ambisome OR posaconazole OR noxafil OR fluconazole OR diflucan OR micafungin OR mycamine OR nystatin OR tropisetron OR antiemetic OR aprepitant OR emend OR fosaprepitant OR ivemend OR granisetron OR kytril OR ondansetron OR zofran OR palonosetron OR aloxi OR metoclopramide OR reglan OR primperan OR dexamethasone OR antiviral OR aciclovir OR zovirax OR ganciclovir OR cytovene OR valganciclovir OR valcyte OR oseltamivir OR tamiflu OR foscarnet OR foscavir OR comedication* OR (co NEXT medication*) OR (supportive care NEXT medication*))
**EMBASE**
('ototoxicity'/exp OR 'ototox*':ti,ab,kw OR 'otol*':ti,ab,kw OR 'cochleotox*':ti,ab,kw OR 'vestibulotox*':ti,ab,kw OR 'cochlear toxic*':ti,ab,kw OR 'vestibular toxic*':ti,ab,kw OR 'tinnitus'/exp OR 'tinnitus':ti,ab,kw OR 'hearing imp*':ti,ab,kw OR 'hearing impairment'/exp OR 'hearing loss*':ti,ab,kw OR 'hearing':ti,ab,kw OR 'deaf*':ti,ab,kw OR 'vertigo'/exp OR 'vertigo':ti,ab,kw OR 'dizz*':ti,ab,kw OR 'audio*':ti,ab,kw) AND ('cisplatin':ti,ab,kw OR 'platinum':ti,ab,kw OR 'platinum*':ti,ab,kw OR 'platin':ti,ab,kw) AND ('pediatric':ti,ab,kw OR 'child*':ti,ab,kw OR 'paediatric':ti,ab,kw) AND ('vincristine' OR 'vinblastine' OR 'cyclophosphamide' OR 'ifosfamide' OR 'etoposide' OR 'doxorubicin' OR 'fluorouracil' OR 'fluorouracil'/exp OR 'hd-mtx' OR 'methotrexate' OR 'lomustine' OR 'aminoglyco*' OR 'glycopep*' OR 'vancomycin' OR 'teicoplanin' OR 'tobramycin' OR 'gentamycin*' OR 'amikacin' OR 'diuretic*' OR 'furosemide' OR 'lasix' OR 'spironolactone' OR 'aldactone' OR 'hydrochlorothiazide' OR 'microzide' OR 'bumetanide' OR 'bumex' OR 'burinex' OR 'antifungal' OR 'amphotericin*' OR 'fungizone' OR 'ambisome' OR 'posaconazole' OR 'noxafil' OR 'fluconazole' OR 'diflucan' OR 'micafungin' OR 'mycamine' OR 'nystatin' OR 'tropisetron' OR 'antiemetic' OR 'aprepitant' OR 'emend' OR 'fosaprepitant' OR 'ivemend' OR 'ivemend' OR 'granisetron' OR 'kytril' OR 'ondansetron' OR 'zofran' OR 'palonosetron' OR 'aloxi' OR 'metoclopramide' OR 'reglan' OR 'primperan' OR 'dexamethasone' OR 'antiviral' OR 'aciclovir' OR 'zovirax' OR 'ganciclovir' OR 'cytovene' OR 'valganciclovir' OR 'valcyte' OR 'oseltamivir' OR 'tamiflu' OR 'foscarnet' OR 'foscavir' OR 'comedication*' OR 'co-medication*' OR 'supportive care medication*')
**Pubmed (MEDLINE)**
("ototoxicity"[MeSH Terms] OR "ototox*"[Title/Abstract] OR "otol*"[Title/Abstract] OR "cochleotox*"[Title/Abstract] OR "vestibulotox*"[Title/Abstract] OR "cochlear toxic*"[Title/Abstract] OR "vestibular toxic*"[Title/Abstract] OR "tinnitus"[MeSH Terms] OR "tinnitus"[Title/Abstract] OR "hearing imp*"[Title/Abstract] OR "hearing loss"[MeSH Terms] OR "hearing loss*"[Title/Abstract] OR "hearing"[Title/Abstract] OR "deaf*"[Title/Abstract] OR "vertigo"[MeSH Terms] OR "vertigo"[Title/Abstract] OR "dizz*"[Title/Abstract] OR "audio*"[Title/Abstract]) AND ("cisplatin"[Title/Abstract] OR "platinum"[Title/Abstract] OR "platinum*"[Title/Abstract] OR "platin"[Title/Abstract]) AND ("pediatric"[Title/Abstract] OR "child*"[Title/Abstract] OR "paediatric"[Title/Abstract]) AND ("vincristine"[All fields] OR "vinblastine"[All fields] OR "cyclophosphamide"[All fields] OR "ifosfamide"[All fields] OR "etoposide"[All fields] OR "doxorubicin"[All fields] OR "fluorouracil"[All fields] OR "Fluorouracil"[Mesh] OR "HD-MTX"[All fields] OR "methotrexate"[All fields] OR "lomustine"[All fields] OR "aminoglyco*"[All fields] OR "glycopep*"[All fields] OR "vancomycin"[All fields] OR "teicoplanin"[All fields] OR "tobramycin"[All fields] OR "gentamycin*"[All fields] OR "amikacin"[All fields] OR "diuretic*"[All fields] OR "furosemide"[All fields] OR "lasix"[All fields] OR "spironolactone"[All fields] OR "aldactone"[All fields] OR "hydrochlorothiazide"[All fields] OR "microzide"[All fields] OR "bumetanide"[All fields] OR "bumex"[All fields] OR "burinex"[All fields] OR "antifungal"[All fields] OR "amphotericin*"[All fields] OR "fungizone"[All fields] OR "ambisome"[All fields] OR "posaconazole"[All fields] OR "noxafil"[All fields] OR "fluconazole"[All fields] OR "diflucan"[All fields] OR "micafungin"[All fields] OR "mycamine"[All fields] OR "nystatin"[All fields] OR "tropisetron"[All fields] OR "antiemetic"[All fields] OR "aprepitant"[All fields] OR "emend"[All fields] OR "fosaprepitant"[All fields] OR "ivemend"[All fields] OR "ivemend"[All fields] OR "granisetron"[All fields] OR "kytril"[All fields] OR "ondansetron"[All fields] OR "zofran"[All fields] OR "palonosetron"[All fields] OR "aloxi"[All fields] OR "metoclopramide"[All fields] OR "reglan"[All fields] OR "primperan"[All fields] OR "dexamethasone"[All fields] OR "antiviral"[All fields] OR "aciclovir"[All fields] OR "zovirax"[All fields] OR "ganciclovir"[All fields] OR "cytovene"[All fields] OR "valganciclovir"[All fields] OR "valcyte"[All fields] OR "oseltamivir"[All fields] OR "tamiflu"[All fields] OR "foscarnet"[All fields] OR "foscavir"[All fields] OR "comedication*"[All fields] OR "co-medication*"[All fields] OR "supportive care medication*"[All fields])

Supplementary Table 2: The odds ratios of the risk developing cisplatin-induced hearing loss of the studies including possible confounding factors

Author and year of publication	OR method	Study details		Cisplatin treatment		Cisplatin + comedication		Outcome		Possible confounding factors
		Type of comedication	Hearing loss classification system	No HL	HL	No HL	HL	Type OR	OR (95% CI)	
**Vincristine**										
Diepstraten, 2024 (1)	UVA	Vincristine	Muenster (grade ≥2b)	NA	NA	NA	NA	Unadjusted OR	1.85 (1.03-3.30)	ENT surgery, H&N RT, carboplatin, oxaliplatin, vancomycin, teicoplanin, amplodipine, furosemide
Diepstraten, 2024 (1)	UVA	Vincristine	SIOP (grade ≥2)	NA	NA	NA	NA	Unadjusted OR	2.60 (1.45-4.69)	ENT surgery, H&N RT, carboplatin, oxaliplatin, vancomycin, teicoplanin, amplodipine, furosemide
Siemens, 2023 (2)	Prevalence	Vincristine	CTCAE v5 (≥2)	111	106	23	131	Crude OR	5.96 (3.56-10.00)	Cranial irradiation, furosemide, gentamycin, tobramycin
Strebel, 2023 (3)	UVA	Vincristine	SIOP (grade ≥2)	NA	NA	NA	NA	Unadjusted OR	3.50 (2.00-6.00)	Cranial radiotherapy, stem cell transplantation, carboplatin
Meijer, 2022 (4)	UVA	Vincristine	SIOP (grade ≥2)	NA	NA	NA	NA	Unadjusted HR*	2.89 (2.08-4.02)	Carboplatin, cranial irradiation, tobramycin, gentamycin, vancomycin, furosemide
Moke, 2021 (5)	UVA[1]	Vincristine	SIOP (grade ≥2)	NA	NA	NA	NA	Unadjusted OR	5.77 (4.34-7.71)	Carboplatin, cranial radiation, stem cell transplantation or rescue, VP shunt
Vos, 2016 (6)	Prevalence	Vincristine	Chang (grade >0)	77	70	2	7	Crude OR	3.85 (0.77-19.15)	Carboplatin, genetic variants
Castelán-Martínez, 2014 (7)	UVA	Vincristine	CTCAE v4.03 (grade ≥1)	NA	NA	NA	NA	Unadjusted OR	1.70 (0.38-7.58)	Doxorubicin, ifosfamide, cyclophaphamide, etoposide, amikacin
Hagleitner, 2015 (8)	Prevalence	Vincristine	CTCAE v3 (grade ≥2)<br>SIOP (grade ≥2)	84	53	6	5	Crude OR	1.32 (0.38-4.54)	Aminoglycosides, genetic variants
Pussegoda, 2013 (9)	Prevalence	Vincristine	CTCAE v3 (grade ≥2)	95	29	81	112	Crude OR	4.53 (2.73-7.50)	Cranial irradiation, tobramycin, gentamycin, vancomycin, genetic variants in TPMT
Ross, 2009 (10)	Prevalence	Vincristine	CTCAE (grade ≥2)	56	97	0	9	Crude OR	11.01 (0.63-192.8)	Cranial, irradiation, gentamycin, tobramycin, vancomycin
**Other chemotherapeutic agents**										
Metwally, 2025 (11)	Prevalence	Cyclophosphamide	CTCAE v5 (grade ≥3)	85	27	54	2	Crude OR	0.12 (0.03-0.51)	Vincristine, lomustine, cisplatin dose, radiotherapy
Becktell, 2024 (12)	Prevalence	Bleomycin, Dactinomycin, cyclophosphamide (MAP vs MABCDP)	CTCAE v4.03 (grade ≥3)	55	236	27	187	Crude OR	0.62 (0.38-1.02)	Methotrexate, doxorubicin, ifosfamide, etoposide, surgery, radiotherapy
Moore, 2023 (13)	Prevalence	Methotrexate	SIOP (grade ≥1)	2	10	1	5	Crude OR	1 (0.07-13.87)	Radiation, carboplatin, ifosfamide, bleomycin, cyclophosphamide, doxorubicin, vincristine, cytarabine

Castelán-Martínez, 2014 (7)	UVA	Doxorubicin	CTCAE v4.03 (grade ≥1)	NA	NA	NA	NA	Unadjusted OR	0.46 (0.15-1.46)	Ifosfamide, cyclophaphamide, etoposide, amikacin, vincristine
Perilongo, 2009 (14)	Prevalence	Doxorubicin	Brock (grade ≥2)	71	18	64	15	Crude OR	0.92 (0.43-1.98)	NA
Packer, 2006 (15)	Prevalence	Cyclophosphamide vs lomustine	(grade ≥3)	L: 139	54	C: 143	43	Crude OR	1.29 (0.81-2.05)	Craniospinal radiotherapy, vincristine
Winkler, 1990 (16)	Prevalence	Ifosfamide vs bleomycin	NA	B: 23	1	I: 22	19	Crude OR	19.86 (2.45-161.3)	Methotrexate, doxorubicin, cyclophosphamide
**Loop diuretics**										
Diepstraten, 2024 (1)	UVA	Furosemide	Muenster (grade ≥2b)	NA	NA	NA	NA	Unadjusted OR	1.54 (0.85-2.77)	ENT surgery, H&N RT, carboplatin, oxaliplatin, gentamicin, vancomycin, teicoplanin, amplodipine, vincristine
Diepstraten, 2024 (1)	UVA	Furosemide	SIOP (grade ≥2)	NA	NA	NA	NA	Unadjusted OR	1.30 (0.72-2.36)	ENT surgery, H&N RT, carboplatin, oxaliplatin, gentamicin, vancomycin, teicoplanin, amplodipine, vincristine
Siemens, 2023 (2)	Prevalence	Furosemide	CTCAE v5 (≥2)	137	235	0	2	Crude OR	2.92 (0.14-61.26)	Cranial irradiation, vincristine, gentamycin, tobramycin
Meijer, 2022 (4)	UVA	Furosemide	SIOP (grade ≥2)	NA	NA	NA	NA	Unadjusted OR	1.26 (0.93-1.72)	Carboplatin, cranial irradiation, tobramycin, gentamycin, vancomycin, vincristine
Clemens, 2016 (17)	UVA	Furosemide	Muenster (grade ≥2b)	NA	NA	NA	NA	Unadjusted OR	2.30 (1.40-3.90)	Carboplatin, vancomycin, tobramycin, gentamicin
Olgun, 2016 (18)	Prevalence	Furosemide	Brock (grade ≥2)	8	1	40	23	Crude OR	4.60 (0.54-39.1)	Cranial irradiation, genetic variants (6 SNPs), carboplatin, aminoglycosides
Olgun, 2016 (18)	Prevalence	Furosemide	Muenster (grade ≥2)	7	2	35	28	Crude OR	2.80 (0.54-14.6)	Cranial irradiation, genetic variants (6 SNPs), carboplatin, aminoglycosides
**Aminoglycosides**										
Diepstraten, 2024 (1)	UVA	Gentamycin	Muenster (grade ≥2b)	NA	NA	NA	NA	Unadjusted OR	2.28 (1.08-4.78)	ENT surgery, H&N RT, carboplatin, oxaliplatin, furosemide, vancomycin, teicoplanin, amplodipine, vincristine
Diepstraten, 2024 (1)	UVA	Gentamycin	SIOP (grade ≥2)	NA	NA	NA	NA	Unadjusted OR	2.17 (1.10-4.31)	ENT surgery, H&N RT, carboplatin, oxaliplatin, furosemide, vancomycin, teicoplanin, amplodipine, vincristine
Romano, 2023 (19)	UVA	Not specified	SIOP (grade >0)	NA	NA	NA	NA	Unadjusted HR*	1.19 (0.42-1.96)	Neurosurgery, cerebral radiotherapy, carboplatin
Siemens, 2023 (2)	Prevalence	Gentamycin	CTCAE v5 (≥2)	133	233	1	4	Crude OR	2.28 (0.25-20.64)	Cranial irradiation, vincristine, furosemide, tobramycin
Siemens, 2023 (2)	Prevalence	Tobramycin	CTCAE v5 (≥2)	130	222	4	15	Crude OR	2.20 (0.71-6.76)	Cranial irradiation, vincristine, furosemide, gentamycin
Sherief, 2022 (20)	Prevalence	Amikacin	Brock (grade ≥2)	44	16	4	0	Crude OR	0.30 (0.02-5.88)	Carboplatin, ototoxic diuretics, genetic variants

Sriyapai, 2022 (21)	Prevalence	Not specified	CTCAE v5 (≥2)	23	19	0	5	Crude OR	13.3 (0.69-255.0)	Cranial radiation, surgical removal, bone marrow transplantation, bleomycin, carboplatin, doxorubicin, etoposide, cyclophosphamide, ifosfamide, methotrexate irinotecan, topotecan, 5-Flurouracil, vincristine
Meijer, 2022 (4)	UVA	Not specified, administered for >30 d	SIOP (grade ≥2)	NA	NA	NA	NA	Unadjusted HR*	1.90 (1.27-3.01)	Carboplatin, cranial irradiation, furosemide, vincristine
Olgun, 2021 (22)	Prevalence	Not specified	Brock (grade ≥2)	45	17	14	8	Crude OR	1.51 (0.54-4.25)	Cranial irradiation, carboplatin, genetic variants
Olgun, 2021 (22)	Prevalence	Not specified	Chang (grade ≥2)	41	21	14	8	Crude OR	1.11 (0.40-3.08)	Cranial irradiation, carboplatin, genetic variants
Olgun, 2021 (22)	Prevalence	Not specified	Muenster (grade ≥2b)	43	19	14	8	Crude OR	1.29 (0.47-3.60)	Cranial irradiation, carboplatin, genetic variants
Turan, 2019 (23)	Prevalence	Not specified	Brock	19	18	9	4	Crude OR	0.47 (0.12-1.80)	Irradiation, etoposide, vincristine, cyclophosphamide, bleomycin, doxorubicin, genetic variants
Clemens, 2016 (17)	UVA	Tobramycin	Muenster (grade ≥2b)	NA	NA	NA	NA	Unadjusted OR	1.30 (0.80-2.40)	Carboplatin, vancomycin, furosemide, gentamicin
Clemens, 2016 (17)	UVA	Gentamycin	Muenster (grade ≥2b)	NA	NA	NA	NA	Unadjusted OR	1.10 (0.70-1.80)	Carboplatin, vancomycin, tobramycin, furosemide
Olgun, 2016 (18)	Prevalence	Not specified	Brock (grade ≥2)	NA	NA	NA	NA	Unadjusted OR	3.84 (1.18-12.47)	Cranial irradiation, genetic variants (6 SNPs), carboplatin, furosemide
Olgun, 2016 (18)	Prevalence	Not specified	Muenster (grade ≥2b)	NA	NA	NA	NA	Unadjusted OR	3.55 (1.18-10.66)	Cranial irradiation, genetic variants (6 SNPs), carboplatin, furosemide
Castelán-Martínez, 2014 (7)	UVA	Amikacin	CTCAE v4 (grade ≥1)	NA	NA	NA	NA	Unadjusted OR	0.77 (0.14-4.15)	Doxorubicin, ifosfamide, cyclophosphamide, etoposide, vincristine
Hagleitner, 2015 (8)	Prevalence	Not specified	CTCAE v3 (grade ≥2)<br>SIOP (grade ≥2)	80	43	10	15	Crude OR	2.79 (1.16-6.74)	Vincristine, genetic variants
Landier, 2014 (24)	UVA	Hospitalization for infection	Brock (grade ≥3)	NA	NA	NA	NA	Adjusted OR	5.1 (1.7-14.9)	Carboplatin, vincristine, etoposide, doxorubicin, cyclophosphamide
Landier, 2014 (25)	UVA	Hospitalization for infection	Chang (grade ≥2b)	NA	NA	NA	NA	Adjusted OR	2.2 (1.2-4.3)	Carboplatin, vincristine, etoposide, doxorubicin, cyclophosphamide
Landier, 2014 (25)	UVA	Hospitalization for infection	CTCAE v3 (grade ≥3)	NA	NA	NA	NA	Adjusted OR	1.8 (0.86-3.7)	Carboplatin, vincristine, etoposide, doxorubicin, cyclophosphamide
Choeyprasert, 2013 (26)	Prevalence	Not specified	Brock (grade ≥1)	5	29	9	25	Crude OR	0.48 (0.14-1.62)	Cranial irradiation, genetic variants
Pussegoda, 2013 (9)	Prevalence	Gentamycin	CTCAE v3 (grade ≥2)	98	26	151	42	Crude OR	1.05 (0.60-1.82)	Cranial irradiation, tobramycin, vincristine, vancomycin, genetic variants in TPMT
Pussegoda, 2013 (9)	Prevalence	Tobramycin	CTCAE v3 (grade ≥2)	94	30	138	55	Crude OR	1.25 (0.75-2.09)	Cranial irradiation, vincristine, gentamycin, vancomycin, genetic variants in TPMT
Lewis, 2009 (27)	Prevalence	Not specified	Brock (grade ≥1)	14	7	7	8	Crude OR	2.29 (0.59-8.91)	Carboplatin, vancomycin, methotrexate, doxorubicin, ifosfamide

Ross, 2009 (10)	Prevalence	Gentamycin	CTCAE (grade ≥2)	53	96	3	10	Crude OR	1.84 (0.49-6.98)	Cranial, irradiation, vincristine, tobramycin, vancomycin
Ross, 2009 (10)	Prevalence	Tobramycin	CTCAE (grade ≥2)	46	88	10	18	Crude OR	0.94 (0.40-2.20)	Cranial, irradiation, vincristine, gentamycin, vancomycin
Kretschmar, 1990 (28)	Prevalence	Not specified	NA	16	4	7	1	Crude OR	0.57 (0.05-6.08)	Radiotherapy, vincristine, mannitol, amphotericin B
**Glycopeptides**										
Diepstraten, 2024 (1)	UVA	Vancomycin	Muenster (grade ≥2b)	NA	NA	NA	NA	Unadjusted OR	3.09 (1.71-5.60)	ENT surgery, H&N RT, carboplatin, oxaliplatin, furosemide, gentamicin, teicoplanin, amlodipine, vincristine
Diepstraten, 2024 (1)	UVA	Vancomycin	SIOP (grade ≥2)	NA	NA	NA	NA	Unadjusted OR	2.71 (1.49-4.92)	ENT surgery, H&N RT, carboplatin, oxaliplatin, furosemide, gentamicin, teicoplanin, amlodipine, vincristine
Diepstraten, 2024 (1)	UVA	Teicoplanin	Muenster (grade ≥2b)	NA	NA	NA	NA	Unadjusted OR	1.66 (0.82-3.36)	ENT surgery, H&N RT, carboplatin, oxaliplatin, furosemide, gentamicin, vancomycin, amlodipine, vincristine
Diepstraten, 2024 (1)	UVA	Teicoplanin	SIOP (grade ≥2)	NA	NA	NA	NA	Unadjusted OR	1.55 (0.80-3.01)	ENT surgery, H&N RT, carboplatin, oxaliplatin, furosemide, gentamicin, vancomycin, amlodipine, vincristine
Clemens, 2016 (17)	UVA	Vancomycin	Muenster (grade ≥2b)	NA	NA	NA	NA	Unadjusted OR	1.30 (0.90-1.90)	Carboplatin, furosemide, tobramycin, gentamicin
Pussegoda, 2013 (9)	Prevalence	Vancomycin	CTCAE v3 (grade ≥2)	99	25	142	51	Crude OR	1.42 (0.83-2.45)	Cranial irradiation, tobramycin, gentamycin, vincristine, genetic variants in TPMT
Lewis, 2009 (27)	Prevalence	Vancomycin	Brock (grade ≥1)	14	7	7	8	Crude OR	2.29 (0.59-8.91)	Carboplatin, aminoglycosides, methotrexate, doxorubicin, ifosfamide
Ross, 2009 (10)	Prevalence	Vancomycin	CTCAE (grade ≥2)	50	93	6	13	Crude OR	1.16 (0.42-3.25)	Cranial, irradiation, vincristine, gentamycin, tobramycin

**HR: hazard ratio instead of odds ratio; [1]Univariable analysis at latest follow-up stratified by patients without cranial radiotherapy.*
C5V: cisplatin/5-fluorouracil/vincristine, CARBO: carboplatin, CDDP: cisplatin, CI: confidence interval, CTCAE: Common terminology criteria for adverse events, ENT: ear-nose-throat, HL: hearing loss, HR: hazard ratio, H&N: Head & neck, NA: Not available/not applicable, OR: odds ratio, RT: radiotherapy, TPMT: thiopurine S-methyltransferase, UVA: univariate analysis, v: version

Supplementary Table 3: an overview of the compounds suggested to induce hearing loss as a single agent (without cisplatin)

Comedication type	Compound	Literature
Chemotherapeutic	Vincristine	(29-33)
Chemotherapeutic	Vinblastine	(34)
Aminoglycosides	Amikacin, gentamicin, tobramycin	(35-38)
Glycopeptides	Vancomycin	(35, 39-41)
Loop diuretics	Furosemide, bumetanide, ethacrynic acid	(42-45)
Antifungal medication	Amphotericin B	(46, 47)
Antiviral medication	Antiviral medication	No strong evidence
Antiemetic medication	Antiemetic medication	No strong evidence

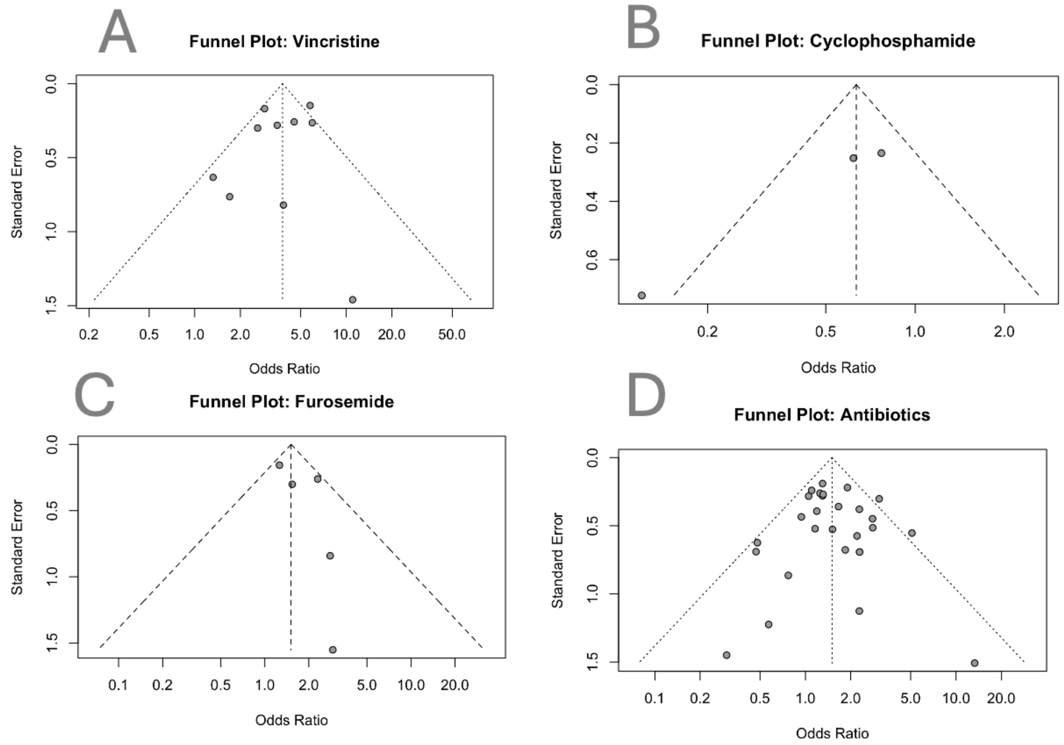

Supplementary Figure 1: funnel plots of total CIHL with A: vincristine, B: cyclophosphamide, C: furosemide, D: aminoglycosides + glycopeptides. Funnel plot showing study precision vs. effect size; asymmetry may suggest publication bias.

Supplementary references

1. Diepstraten FA, Bertram OMM, Helleman HW, Boerboom RA, van Grotel M, Zsíros J, et al. A Retrospective Evaluation of Ototoxicity Monitoring in a Cohort of Pediatric Patients with Solid Tumors, Treated in the Dutch National Cancer Center. *Cancer Rep (Hoboken)* (2024) 7(11):e70046. doi: 10.1002/cnr2.70046.
2. Siemens A, Brooks B, Rassekh SR, Meijer AJM, van den Heuvel-Eibrink MM, Xu W, et al. Role of Cisplatin Dose Intensity and Tpmt Variation in the Development of Hearing Loss in Children. *Ther Drug Monit* (2023) 45(3):345-53. doi: 10.1097/ftd.0000000000001085.
3. Strebel S, Mader L, Jörger P, Waespe N, Uhlmann S, von der Weid N, et al. Hearing Loss after Exposure to Vincristine and Platinum-Based Chemotherapy among Childhood Cancer Survivors. *EJC Paediatric Oncology* (2023) 1:100017. doi: https://doi.org/10.1016/j.ejcped.2023.100017.
4. Meijer AJM, Li KH, Brooks B, Clemens E, Ross CJ, Rassekh SR, et al. The Cumulative Incidence of Cisplatin-Induced Hearing Loss in Young Children Is Higher and Develops at an Early Stage during Therapy Compared with Older Children Based on 2052 Audiological Assessments. *Cancer* (2022) 128(1):169-79. Epub 20210907. doi: 10.1002/cncr.33848.
5. Moke DJ, Luo C, Millstein J, Knight KR, Rassekh SR, Brooks B, et al. Prevalence and Risk Factors for Cisplatin-Induced Hearing Loss in Children, Adolescents, and Young Adults: A Multi-Institutional North American Cohort Study. *Lancet Child Adolesc Health* (2021) 5(4):274-83. Epub 20210212. doi: 10.1016/s2352-4642(21)00020-1.
6. Vos HI, Guchelaar H-J, Gelderblom H, de Bont ESJM, Kremer LCM, Naber AM, et al. Replication of a Genetic Variant in Acyp2 Associated with Cisplatin-Induced Hearing Loss in Patients with Osteosarcoma. *Pharmacogenetics and Genomics* (2016) 26(5).
7. Castelán-Martínez OD, Jiménez-Méndez R, Rodríguez-Islas F, Fierro-Evans M, Vázquez-Gómez BE, Medina-Sansón A, et al. Hearing Loss in Mexican Children Treated with Cisplatin. *International Journal of Pediatric Otorhinolaryngology* (2014) 78(9):1456-60. doi: https://doi.org/10.1016/j.ijporl.2014.06.007.
8. Hagleitner MM, Coenen MJH, Patino-Garcia A, de Bont ESJM, Gonzalez-Neira A, Vos HI, et al. Influence of Genetic Variants in Tpmt and Comt Associated with Cisplatin Induced Hearing Loss in Patients with Cancer: Two New Cohorts and a Meta-Analysis Reveal Significant Heterogeneity between Cohorts. *PLOS ONE* (2015) 9(12):e115869. doi: 10.1371/journal.pone.0115869.
9. Pussegoda K, Ross CJ, Visscher H, Yazdanpanah M, Brooks B, Rassekh SR, et al. Replication of Tpmt and Abcc3 Genetic Variants Highly Associated with Cisplatin-Induced Hearing Loss in Children. *Clin Pharmacol Ther* (2013) 94(2):243-51. Epub 20130410. doi: 10.1038/clpt.2013.80.
10. Ross CJ, Katzov-Eckert H, Dubé MP, Brooks B, Rassekh SR, Barhdadi A, et al. Genetic Variants in Tpmt and Comt Are Associated with Hearing Loss in Children Receiving Cisplatin Chemotherapy. *Nat Genet* (2009) 41(12):1345-9. Epub 20091108. doi: 10.1038/ng.478.
11. Metwally SM, El-Ayadi M, Maher E, El-Minawi MS, Zaghloul MS, Taha H, et al. Impact of Partial Substitution of Cisplatin with Cyclophosphamide on Acute Toxicities in Standard-Risk Medulloblastoma. *Journal of Neuro-Oncology* (2025) 174(3):689-97. doi: 10.1007/s11060-025-05098-7.
12. Becktell K, Chen Y, Yasui Y, Phelan R, Armstrong GT, Link M, et al. Long-Term Outcomes among Survivors of Childhood Osteosarcoma: A Report from the Childhood Cancer Survivor Study (Ccss). *Pediatric Blood and Cancer* (2024) 71(10). doi: 10.1002/pbc.31189.
13. Moore B, Sheets G, Doss J, Umrigar A, Norman M, Fang Z, et al. Is Methotrexate Ototoxic? Investigating the Ototoxic Late Effects of Pediatric Cancer Treatment. *Am J Audiol* (2023) 32(3):657-64. Epub 20230802. doi: 10.1044/2023_aja-22-00157.

14. Perilongo G, Maibach R, Shafford E, Brugieres L, Brock P, Morland B, et al. Cisplatin Versus Cisplatin Plus Doxorubicin for Standard-Risk Hepatoblastoma. *New England Journal of Medicine* (2009) 361(17):1662-70. doi: 10.1056/NEJMoa0810613.
15. Packer RJ, Gajjar A, Vezina G, Rorke-Adams L, Burger PC, Robertson PL, et al. Phase Iii Study of Craniospinal Radiation Therapy Followed by Adjuvant Chemotherapy for Newly Diagnosed Average-Risk Medulloblastoma. *Journal of clinical oncology* (2006) 24(25):4202-8. doi: 10.1200/JCO.2006.06.4980.
16. Winkler K, Bielack S, Delling G, Salzer-Kuntschik M, Kotz R, Greenshaw C, et al. Effect of Intraarterial Versus Intravenous Cisplatin in Addition to Systemic Doxorubicin, High-Dose Methotrexate, and Ifosfamide on Histologic Tumor Response in Osteosarcoma (Study Coss-86). *Cancer* (1990) 66(8):1703-10. doi: 10.1002/1097-0142(19901015)66:8<1703::aid-cncr2820660809>3.0.co;2-v.
17. Clemens E, de Vries AC, Pluijm SF, am Zehnhoff-Dinnesen A, Tissing WJ, Loonen JJ, et al. Determinants of Ototoxicity in 451 Platinum-Treated Dutch Survivors of Childhood Cancer: A Dcog Late-Effects Study. *European Journal of Cancer* (2016) 69:77-85. doi: 10.1016/j.ejca.2016.09.023.
18. Olgun Y, Aktaş S, Altun Z, Kırkım G, Kızmazoğlu D, Erçetin AP, et al. Analysis of Genetic and Non Genetic Risk Factors for Cisplatin Ototoxicity in Pediatric Patients. *Int J Pediatr Otorhinolaryngol* (2016) 90:64-9. Epub 20160904. doi: 10.1016/j.ijporl.2016.09.001.
19. Romano A, Rivetti S, Brigato F, Mastrangelo S, Attinà G, Maurizi P, et al. Early and Long-Term Ototoxicity Noted in Children Due to Platinum Compounds: Prevalence and Risk Factors. *Biomedicines* [Internet]. (2023; 11(2).
20. Sherief LM, Rifky E, Attia M, Ahmed R, Kamal NM, Oshi MAM, et al. Platinum-Induced Ototoxicity in Pediatric Cancer Survivors: Gstp1 C.313a>G Variant Association. *Medicine (Baltimore)* (2022) 101(45):e31627. doi: 10.1097/md.0000000000031627.
21. Sriyapai T, Thongyai K, Phuakpet K, Vathana N, Buaboonnam J, Sanpakit K. Ototoxicity and Long-Term Hearing Outcome in Pediatric Patients Receiving Cisplatin. *Turk J Pediatr* (2022) 64(3):531-41. doi: 10.24953/turkjped.2021.5012.
22. Olgun Y, Çakir Kizmazoğlu D, İnce D, Ellidokuz H, Güneri EA, Olgun N, et al. Evaluation of Risk Factors Causing Ototoxicity in Childhood Cancers Located in the Head and Neck Region Treated with Platinum-Based Chemotherapy. *J Pediatr Hematol Oncol* (2021) 43(7):e930-e4. doi: 10.1097/mph.0000000000002158.
23. Turan C, Kantar M, Aktan Ç, Kosova B, Orman M, Bilgen C, et al. Cisplatin Ototoxicity in Children: Risk Factors and Its Relationship with Polymorphisms of DNA Repair Genes Ercc1, Ercc2, and Xrcc1. *Cancer Chemotherapy and Pharmacology* (2019) 84(6):1333-8. doi: 10.1007/s00280-019-03968-2.
24. Landier W. Ototoxicity and Cancer Therapy. *Cancer* (2016) 122(11):1647-58.
25. Landier W, Knight K, Wong FL, Lee J, Thomas O, Kim H, et al. Ototoxicity in Children with High-Risk Neuroblastoma: Prevalence, Risk Factors, and Concordance of Grading Scales—a Report from the Children's Oncology Group. *Journal of Clinical Oncology* (2014) 32(6):527-34. doi: 10.1200/JCO.2013.51.2038.
26. Choeyprasert W, Sawangpanich R, Lertsukprasert K, Udomsubpayakul U, Songdej D, Unurathapan U, et al. Cisplatin-Induced Ototoxicity in Pediatric Solid Tumors: The Role of Glutathione S-Transferases and Megalin Genetic Polymorphisms. *J Pediatr Hematol Oncol* (2013) 35(4):e138-43. doi: 10.1097/MPH.0b013e3182707fc5.
27. Lewis MJ, DuBois SG, Fligor B, Li X, Goorin A, Grier HE. Ototoxicity in Children Treated for Osteosarcoma. *Pediatr Blood Cancer* (2009) 52(3):387-91. doi: 10.1002/pbc.21875.

28. Kretschmar CS, Warren MP, Lavally BL, Dyer S, Tarbell NJ. Ototoxicity of Preradiation Cisplatin for Children with Central Nervous System Tumors. *Journal of Clinical Oncology* (1990) 8(7):1191-8. doi: 10.1200/jco.1990.8.7.1191.
29. Lugassy G, Shapira A. Sensorineural Hearing Loss Associated with Vincristine Treatment. *Blut* (1990) 61:320-1.
30. Aydogdu I, O. O, I. K, E. K, A. S, and Yildiz R. Bilateral Transient Hearing Loss Associated with Vincristine Therapy: Case Report. *Journal of Chemotherapy* (2000) 12(6):530-2. doi: 10.1179/joc.2000.12.6.530.
31. Yousif H, Richardson SG, Saunders WA. Partially Reversible Nerve Deafness Due to Vincristine. *Postgrad Med J* (1990) 66(778):688-9. doi: 10.1136/pgmj.66.778.688.
32. Riga M, Psarommatis I, Korres S, Lyra C, Papadeas E, Varvutsi M, et al. The Effect of Treatment with Vincristine on Transient Evoked and Distortion Product Otoacoustic Emissions. *International Journal of Pediatric Otorhinolaryngology* (2006) 70(6):1003-8. doi: https://doi.org/10.1016/j.ijporl.2005.10.011.
33. Rao S, Kumar R, Bhat J, Kamath N. Does Vincristine Affect Cochlear Function in Children with Acute Lymphoblastic Leukaemia? *Bangladesh Journal of Medicine* (2016) 27(1):3-7.
34. Moss PE, Hickman S, Harrison BR. Ototoxicity Associated with Vinblastine. *Annals of Pharmacotherapy* (1999) 33(4):423-5. doi: 10.1345/aph.18288.
35. Diepstraten FA, Hoetink AE, van Grotel M, Huitema ADR, Stokroos RJ, van den Heuvel-Eibrink MM, et al. Aminoglycoside- and Glycopeptide-Induced Ototoxicity in Children: A Systematic Review. *JAC Antimicrob Resist* (2021) 3(4):dlab184. Epub 20211214. doi: 10.1093/jacamr/dlab184.
36. Jiang M, Karasawa T, Steyger PS. Aminoglycoside-Induced Cochleotoxicity: A Review. *Frontiers in cellular neuroscience* (2017) 11:308.
37. Moore RD, Smith CR, Lietman PS. Risk Factors for the Development of Auditory Toxicity in Patients Receiving Aminoglycosides. *Journal of Infectious Diseases* (1984) 149(1):23-30.
38. Rizzi MD, Hirose K. Aminoglycoside Ototoxicity. *Curr Opin Otolaryngol Head Neck Surg* (2007) 15(5):352-7. doi: 10.1097/MOO.0b013e3282ef772d.
39. Forouzesh A, Moise PA, Sakoulas G. Vancomycin Ototoxicity: A Reevaluation in an Era of Increasing Doses. *Antimicrobial agents and chemotherapy* (2009) 53(2):483-6.
40. Bailie GR, Neal D. Vancomycin Ototoxicity and Nephrotoxicity. *Medical Toxicology and Adverse Drug Experience* (1988) 3(5):376-86. doi: 10.1007/BF03259891.
41. Marissen J, Fortmann I, Humberg A, Rausch TK, Simon A, Stein A, et al. Vancomycin-Induced Ototoxicity in Very-Low-Birthweight Infants. *Journal of Antimicrobial Chemotherapy* (2020) 75(8):2291-8. doi: 10.1093/jac/dkaa156.
42. Schwartz GH, David DS, Riggio RR, Stenzel KH, Rubin AL. Ototoxicity Induced by Furosemide. *New England Journal of Medicine* (1970) 282(25):1413-4.
43. Rybak LP. Ototoxicity of Loop Diuretics#. *Otolaryngologic Clinics of North America* (1993) 26(5):829-44. doi: https://doi.org/10.1016/S0030-6665(20)30770-2.
44. Ding D, Liu H, Qi W, Jiang H, Li Y, Wu X, et al. Ototoxic Effects and Mechanisms of Loop Diuretics. *J Otol* (2016) 11(4):145-56. Epub 20161027. doi: 10.1016/j.joto.2016.10.001.
45. Ikeda K, Oshima T, Hidaka H, Takasaka T. Molecular and Clinical Implications of Loop Diuretic Ototoxicity. *Hearing Research* (1997) 107(1):1-8. doi: https://doi.org/10.1016/S0378-5955(97)00009-9.
46. Ramu R, Sharma B, Karunakara D, Paliwal P, Bansal N, Taneja RS. Liposomal Amphotericin B-Induced Reversible Ototoxicity in a Patient with Disseminated Histoplasmosis. *Indian Journal of Pharmacology* (2021) 53(2):157-9.
47. Munguia R, Daniel SJ. Ototopical Antifungals and Otomycosis: A Review. *International journal of pediatric otorhinolaryngology* (2008) 72(4):453-9.
